# Dose-Escalated SBRT for Borderline and Locally Advanced Pancreatic Cancer: Resectability Rate and Pathological Results of a Multicenter Prospective Study

**DOI:** 10.3390/cancers17020191

**Published:** 2025-01-09

**Authors:** Barbara Salas-Salas, Laura Ferrera-Alayon, Alberto Espinosa-Lopez, Maria Luisa Perez-Rodriguez, Antonio Alayón Afonso, Andres Vera-Rosas, Gabriel Garcia-Plaza, Rodolfo Chicas-Sett, Maria Soledad Martinez-Martin, Elisa Salcedo, Andrea Kannemann, Marta Lloret-Saez-Bravo, Pedro C. Lara

**Affiliations:** 1 Department of Radiation Oncology, University Hospital Dr Negrín Las Palmas de Gran Canaria, Barranco de la Ballena s/n, 35010 Las Palmas de Gran Canaria, Spain; bsalsal@gobiernodecanarias.org (B.S.-S.); lferala@gobiernodecanarias.org (L.F.-A.); marialuisapr96@gmail.com (M.L.P.-R.); aalaafo@gobiernodecanarias.org (A.A.A.); averrosiyx@gobiernodecanarias.org (A.V.-R.); fsalari@gobiernodecanarias.org (E.S.); akannem@gobiernodecanarias.org (A.K.); mllosae@gobiernodecanarias.org (M.L.-S.-B.); 2 Department of Radiation Oncology, University Hospital Virgen de la Arrixaca, Carretera Madrid-Cartagena, S/N, 30120 El Palmar (Murcia), Spain; alopez@murciasalud.es; 3 Hepatic and Pancreatobiliary Surgery Unit, Complejo Hospitalario Universitario Insular Materno Infantil de Gran Canaria, 35001 Las Palmas de Gran Canaria, Spain; ggarpla@gobiernodecanarias.org; 4 Department of Radiation Oncology, ASCIRES GRUPO BIOMEDICO, 46004 Valencia, Spain; rchicas@ufm.edu; 5 Department of Patological Anatomy, Complejo Hospitalario Universitario Insular Materno Infantil de Gran Canaria, 35016 Las Palmas de Gran Canaria, Spain; 6 Canarian Insitute for Cancer Research, 380204 San Cristobal de La Laguna, Spain; 7 Canarian Comprehensive Cancer Center, Department of Oncology, University Hospital San Roque, C. Dolores de la Rocha, 5, 35001 Las Palmas de Gran Canaria, Spain; 8 Department of Medicine, Fernando Pessoa Canarias University, Calle la Juventud, s/n, 35450 Santa Maria de Guía, Spain

**Keywords:** pancreas, cancer, escalated SBRT, pathological response, margins

## Abstract

Objective: We demonstrated for the first time the safety and feasibility of escalating up to 55 Gy/11 Gy/fr/5fr in borderline (BRPC)/unresectable locally advanced pancreatic cancer (LAPC), using the standard LINAC platform. The aim of the present study is to assess for the first time the impact of this high-dose neoadjuvant stereotactic ablative radiotherapy (SABRT) protocol on tumor resectability and pathological responses. Materials/Methods: From June 2017 to December 2022, patients with BRPC/LAPC were treated with neoadjuvant chemotherapy (ChT) and SABRT-escalated doses of SIB at 45 Gy, 50 Gy, and up to 55 Gy (BED ≥ 100). Radiological evaluation was conducted with a CT scan 6-8 weeks post-treatment to determine resectability status based on established criteria (SAR/APA2014). Surgical decisions were made by the multidisciplinary tumor board of the participating institutions. Pathological assessments post-surgery used criteria from the College of American Pathologists (CAP), categorizing resection status as R0 (negative margins), R1 (microscopic tumor margins), and R2 (macroscopic tumor margins). Tumor response was evaluated with the Tumor Response Scoring (TRS) system, as G0 (no viable cancer cells), G1 (single cells or rare small groups), G2 (residual cancer with evident regression), and G3 (extensive residual cancer). Results: Thirty-three patients (p) were included: 39.4% (13p) BRPC/60.6% (20p) LAPC. After ChT-SABRT, 45.5% (15p) were considered resectable, with 11/13 (84.6%) BRPC and 4/20 (20%) LAPC (*p* < 0.0001). One patient refused surgery and other patient died of COVID sepsis. Two more patients had disseminated disease at surgery. Among the 11 patients who underwent full surgery, all patients achieved either clean margins R0: 72.7% (8p) or microscopic affected margins R1: 27.3% (3p). TRS scores were G1: 27.3% (3p), G2: 54.5% (6p), and G3: 18.2% (2p). The present follow-up (FUP) was closed on 1 November 2024 (23.55 months, range: 6–71 months). The mean freedom from local progression as the first cause of disease failure was 43.30 ± 3.09 (37.23–49.38), and the median was not reached. The actuarial 1- and 2-year rates for freedom from local relapse as a first cause of disease failure were 92.3% (87.7–93.3%) and 79.7% (79.7–87.7%), respectively. Conclusions: Neoadjuvant ChT-SABRT in LAPC improves resectability rates and induces relevant tumor regression. These promising findings should be validated by larger sample sizes and extended follow-up.

## Simple Summary

We have already demonstrated that an SBRT-escalated protocol allows for very high doses per fraction (11Gy/fraction), up to total dose up to 55Gy, which is safe and feasible in a standard LINAC-platform for patients with borderline resectable pancreatic cancer (BRPC) or locally advanced pancreatic cancer (LAPC). In this study, we demonstrated for the first time that after neoadjuvant Cht and escalated dose SBRT, resection was indicated in 84.6% of BRPC and 20% of LAPC. Furthermore, all evaluable patients after surgery had complete or minimal disease resection (R0/R1), with objective pathological responses of 81.8% (TRS0-2). The present follow-up (FUP) was closed on 1 November 2024. The actuarial 1- and 2-year rates for freedom from local relapse as a first cause of disease failure were 92.3% (87.7–93.3%) and 79.7% (79.7–87.7%), respectively. This study shows that neoadjuvant ChT-SBRT, particularly with BED > 100, is highly effective in converting BRPC/LAPC to resectable status, providing a pathway to potentially curative surgery in a subset of patients.

## 1. Introduction

Pancreatic cancer is a highly aggressive disease with a 5-year overall survival (OS) rate of only 12% across all stages [1]. Although it accounts for just 3% of cancer cases, it remains the third leading cause of cancer-related deaths in the United States and Europe [1]. Treatment strategies for localized pancreatic cancer depend largely on resectability status, which is determined by the tumor’s relationship with adjacent vascular structures [2].

The role of radiotherapy (RT) in pancreatic cancer has been debated for decades, and its impact on survival remains controversial. While chemoradiotherapy (CRT) is a widely used approach, recent randomized trials have reported conflicting outcomes, showing limited improvements in OS despite achieving better local control (LC) and higher rates of margin-negative resections [3,4,5]. The persistent challenge lies in the high incidence of distant metastasis and local progression, which continue to limit survival outcomes, even with more effective chemotherapies such as mFOLFIRINOX and gemcitabine+nab-paclitaxel [6].

In this context, recent advances in radiotherapy, particularly stereotactic body radiotherapy (SBRT), have introduced a new opportunity to escalate doses safely and improve tumor control [5,7]. SBRT delivers high doses in a reduced number of fractions, minimizing treatment margins and allowing for a better balance between efficacy and safety [8,9]. Current evidence suggests that SBRT, when combined with systemic therapy, can prolong survival in locally advanced pancreatic cancer (LAPC) compared to chemotherapy or conventional fractionated RT alone [10,11,12].

However, defining the optimal SBRT dose remains challenging. Doses between 30 and 45 Gy/3 fractions or 25–50 Gy/5 fractions are commonly used, with the most appropriate regimen still under discussion [13,14,15]. Higher biologically effective doses (BEDs) are essential for achieving long-term tumor control, but proximity to critical organs such as the duodenum, stomach, and small intestine often limits dose escalation [16,17]. To overcome these constraints, techniques such as simultaneous integrated boost (SIB), motion management strategies (e.g., 4D-CT, fiducials, and DIBH), and real-time adaptive planning have been developed [18,19,20,21,22,23,24,25,26].

The introduction of MR-guided radiotherapy (MRgRT) represents a significant step forward in this clinical setting, providing superior soft tissue delineation, real-time monitoring, and motion control. These advantages have enabled safe dose escalation up to 50 Gy or higher while maintaining acceptable toxicity levels and achieving promising clinical results [27,28,29,30,31,32].

Resectability rates following neoadjuvant SBRT are particularly encouraging, reaching up to 50% in some studies [33,34,35], compared to the 20–40% observed with conventional radiotherapy [33]. This improved resectability, combined with chemotherapy, has been shown to increase the likelihood of R0 resections, improve local control, and enhance survival outcomes [12,36,37,38,39].

The aim of the present study is to analyze for the first time the radiological resectability, pathological response, surgical margins, and tumor downstaging in our escalating dose multicenter prospective trial. Furthermore, we detail their pathological outcomes, contributing to a deeper understanding of the impact of ablative dose escalation on this challenging patient population.

## 2. Materials and Methods

### 2.1. Study Population

The patient cohort and treatment protocols for this study were previously described in our earlier publication [40]. In short, patients diagnosed with BRPC and LAPC, eligible for neoadjuvant chemotherapy and local radiation treatment, were included in that prospective study. Patients were diagnosed and treated at four university hospitals of the province of Las Palmas (Canary Islands, Spain). Cancer staging was performed according to the eight edition of the TNM classification system [41].

In short, after the institution indicated neoadjuvant chemotherapy, the eligible patients were referred to SBRT treatment in escalated doses (45 Gy/5 fractions, 50 Gy/5 fractions, 55 Gy/5 fractions) under standard simulation and planning protocols [42,43] and recommendations of movement control systems [44,45]. Metallic endoprostheses [45,46] were used as fiducials. We used the Timmerman constraints for five fractions [47] to evaluate the limiting doses to the OARs.

Later on, patients underwent re-evaluation by the MTB. To evaluate the post-neoadjuvant status, radiological assessment was performed using a CT scan 6–8 weeks after neoadjuvant treatments to determine resectability based on established criteria from the Society of Abdominal Radiology/American Pancreatic Association (SAR/APA 2014) [48].

Tumor resectabiliaty decisions were made by a multidisciplinary tumor board (MTB) from the participating institutions. The assessment of resectability during reevaluation followed the criteria outlined in the NCCN Guidelines, version 3.2024 [49]. Post-surgical margin status was evaluated using the classification system of the College of American Pathologists (CAP) [50]. Also, pathological tumor response was assessed using the Tumor Response Scoring (TRS) system from CAP [51].

### 2.2. Study End Points

The aim of the present study was to analyze for the first time radiological resectability, established by the Society of Abdominal Radiology/American Pancreatic Association (SAR/APA 2014) [48], and the subsequent referral for surgery according to the guidelines outlined in the NCCN Guidelines version 3.2024 [49] in patients receiving escalated SBRT doses up to 55 Gy in 5 fractions. Also, pathological response, surgical margins, and tumor downstaging related to the BED dose administered in our escalating dose multicenter prospective trial were assessed by the TRS system.

### 2.3. Statistical Analysis

We performed the statistical analyses using IBM SPPSS Statistics, version 26 (IBM Corp., Armonk, NY, USA). The chi-squared test was employed to analyze statistical differences in discrete variables and the chi-squared Pearson test to evaluate an association between two categorical variables. In the subgroup analysis, due to the small sample size, Fisher’s exact test was used. Survival figures were generated using the Kaplan–Meier tables. A *p*-value less than 0.05 was considered statistically significant.

## 3. Results

As previously described [40], between June 2017 and December 2022, thirteen patients (39.4%) initially were diagnosed with BRPC and twenty patients (60.6%) with unresectable LAPC (Table 1).

**Figure 1:**
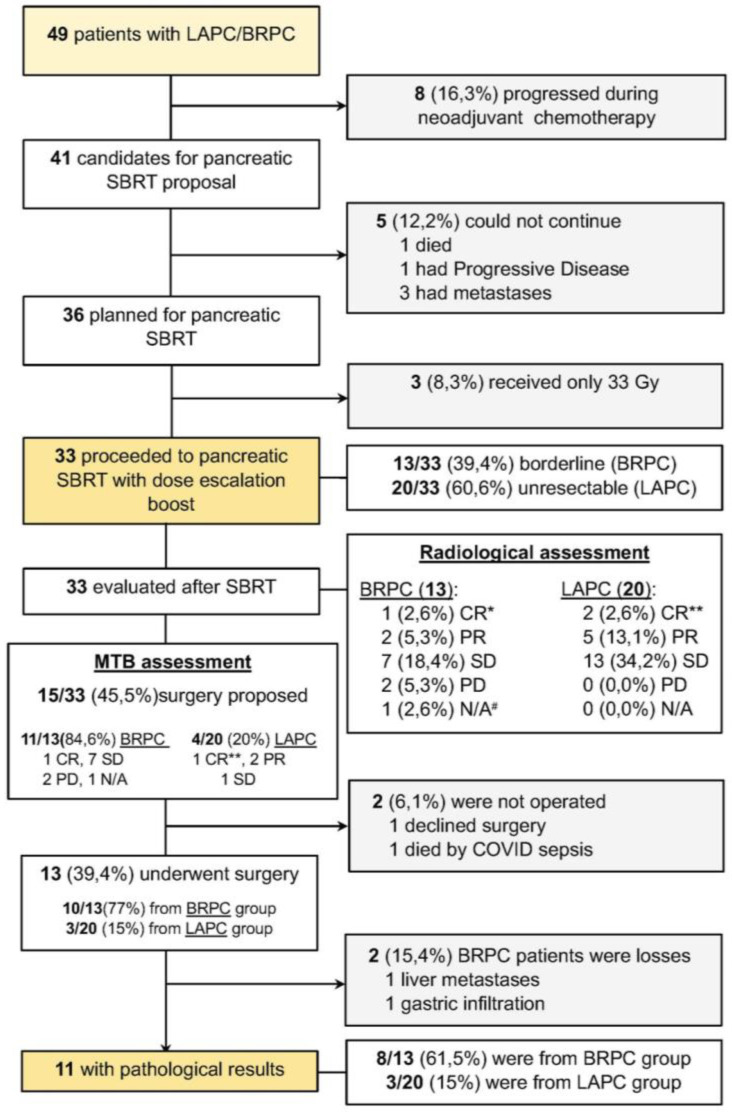
Study flowchart, neoadjuvant SBRT treatment and results. The diagram shows the flow of patients and the dropout of them for various reasons. In addition, the complete flow shows from patient eligibility, proposed neoadjuvant SBRT treatment, pre- and post-treatment resection characteristics to pathological outcomes of patients who underwent surgery and were cM0. CR, complete Response; PR, Partial Response; SD, Stable Disease; PD, Progressive Disease; N/A Does Not Apply; MTB, Multidisciplinary Tumour Board; * Evaluated by CT scan; ** Evaluated by PET-CT; ^#^ Not evaluable due to lack of CT scan prior to surgery.

**Figure 2:**
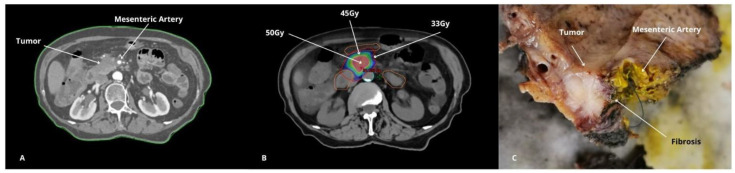
Dose-escalated SBRT effects in borderline resectable pancreatic cancer (BRPC) in an example case: (**A**) CT 3 months post-ChT treatment (tumor >180° in Mesenteric Artery). (**B**) CT with SBRT treatment. (**C**) Surgical specimen showing fibrosis between tumor and vessel.

After ChT-SBRT, fifteen patients (45.45%) were considerate resectable by MTB (Figure 1). Unfortunately, two patients were not operated on due to a death by COVID sepsis and another patient refusing surgery. Ten out of the thirteen (77%) BRPC and 3/20 (15%) LAPC patients were operated on (Figure 2). Two patients in the BRPC group were deemed to finally not to have pancreatic localized cancer, as intraoperatively gastric infiltration in one case and liver metastases in other patient were shown. Finally, in 8/13 (61.5%) of the BRPC and in 3/20 (15%) of the LAPC patients, a pathology report after neoadjuvant treatment was available.

All patients achieved R0 (8/11) or R1 resection (3/11). Pathological tumor responses were scored as 1 (near complete response) in three out of eleven patients (27.3%), as score 2 (partial response) in six out of eleven patients (54.5%), and poor or no response (score 3) in 2/11 patients (18.2%). The aggregated objective response was observed in 9/11 patients (81.8%) (Table 2).

The resectability outcomes after neoadjuvant therapy were strongly related to pretreatment tumor extension (Table 2). In fact, eleven out of the thirteen BRPC patients and 4/20 (20%) of the LAPC patients (*p* < 0.0001) were considered resectable (Table 3). Resectability was also evaluated regarding the biologically effective dose (BED) stratification. As previously published [39], nine patients received a BED dose < 100 Gy and 24 patients received a BED dose ≥ 100 Gy. Among the 33 patients analyzed, 54% (13/24) of those treated with a BED ≥ 100 were deemed resectable compared to only 22.3% (2/9) in the BED < 100 group. Although a higher proportion of resectable cases was observed with BED ≥ 100, the association was not statistically significant (*p* = 0.101). The results suggest a possible trend of an association favoring BED ≥ 100 in improving surgical resectability, highlighting the potential impact of ablative-dose SBRT in enhancing surgical outcomes for BRPC and LAPC previously considered unresectable. The analysis of tumor regression scores (TRSs) grouped by the biologically effective dose (BED) showed no significant association between BED and TRS outcomes or surgical margins.

The present follow-up (FUP) was closed on 1 November 2024. The mean FUP from diagnosis was 23.55 months (range: 6–71 months). The median OS was 19 months, and the actuarial 1- and 2-year OS rates were 63.6% (95% CI 54.5–66.7%) and 27.3% (95% CI (21.2–30.3%), respectively. The mean freedom from local progression as a first cause of disease failure was 43.30 ± 3.09 (37.23–49.38), and the median was not reached. The actuarial 1- and 2-year rates for freedom from local relapse as a first cause of disease failure were 92.3% (87.7–93.3%) and 79.7% (79.7–87.7%), respectively. Unfortunately, the median freedom from distant metastases (FFDM) was 15 months, and the 1- and 2-year actuarial rates for FFDM were 75.1% (62–81.3%) and 39.5% (31.6–55.3%), respectively.

## 4. Discussion

### 4.1. Feasibility and Safety of Dose-Escalated SBRT

Our results confirm the feasibility and safety of dose-escalated SBRT in patients with borderline resectable pancreatic cancer (BRPC) and locally advanced pancreatic cancer (LAPC). The ablative dose escalation scheme of 55 Gy in five fractions, delivered using widely available LINAC platforms, demonstrated promising rates of local control and radiological resectability. Notably, no patients in our cohort experienced grade 3 or higher acute toxicity, reinforcing the safety profile of this approach. These findings are significant in the context of pancreatic cancer, where local progression (LP) remains a major challenge despite advances in systemic therapies.

### 4.2. Comparisons with the Existing Literature

Our study aligns with prior evidence supporting the role of SBRT in achieving improved outcomes compared to conventional radiotherapy. Previous reports indicate that SBRT regimens delivering 30–45 Gy/three fractions or 25–50 Gy/five fractions are associated with resectability rates of 40–50% [32,33,34]. Our findings are consistent with these results, suggesting that dose escalation may further enhance tumor downstaging and increase the likelihood of achieving R0 resections.

From a clinical perspective, the integration of SBRT with systemic therapies, such as mFOLFIRINOX or gemcitabine-based regimens, has shown clear benefits in improving local control and overall survival [35,36,52]. In our cohort, dose-escalated SBRT following systemic therapy not only demonstrated high radiological resectability but also highlighted the feasibility of combining ablative doses with modern chemotherapy protocols.

### 4.3. Technological Advances in Radiotherapy Delivery

The successful delivery of ablative doses in this study relied on precise treatment planning and motion management strategies. Advanced planning systems, such as EasyPlan and Plan 2 Heat, allowed us to optimize dose delivery while minimizing exposure to organs at risk (OARs) like the duodenum, stomach, and small intestine. Furthermore, motion management techniques, including 4D-CT, fiducial markers, and deep inspiration breath hold (DIBH), were essential for reducing treatment margins and ensuring treatment safety [19,20,21,22,23,24].

Recent developments in MRI-guided radiotherapy (MRgRT) offer further advancements in dose escalation for pancreatic tumors. MRgRT provides superior soft tissue visualization, real-time motion control, and adaptive treatment capabilities, enabling the delivery of doses up to 50 Gy or higher while preserving nearby OARs [29]. This is particularly relevant for tumors in close proximity to critical structures, where dose escalation remains a challenge. However, the limited availability of MRgRT technology, especially in many European centers, highlights the importance of achieving comparable outcomes using standard LINAC platforms, as demonstrated in our study.

### 4.4. Strengths and Limitations

The main strengths of this study include its demonstration of the feasibility and safety of dose-escalated SBRT using standard LINAC platforms. This approach, supported by advanced planning systems and motion management techniques, allowed for the precise delivery of ablative doses while maintaining a favorable toxicity profile. Furthermore, our findings contribute valuable data on radiological resectability and local control, which are critical outcomes in this patient population. Comparisons with the existing literature reinforce the clinical relevance of our results and highlight the role of SBRT in multimodal treatment strategies for pancreatic cancer.

However, we acknowledge certain limitations. First, the single-arm design and lack of a control group limit direct comparisons with other treatment modalities. Second, the relatively small sample size may not capture rare adverse events or allow for robust subgroup analyses. Third, long-term outcomes, such as overall survival and disease-free survival, were not assessed in this study and will require further investigation. Finally, while MRgRT represents a promising tool for dose escalation, its limited availability restricts its widespread application, emphasizing the need for accessible alternatives like the techniques presented here.

## 5. Conclusions

This multicenter prospective study demonstrates that dose-escalated SBRT, delivering 50–55 Gy in five fractions, achieves promising results in radiological resectability and pathological tumor responses for borderline resectable (BRPC) and locally advanced pancreatic cancer (LAPC). These findings support the role of dose-escalated SBRT in improving surgical outcomes within multimodal treatment strategies.

## Figures and Tables

**Table 1:** Baseline patient and tumor characteristics.

Characteristic	Number of Patients (%)
Follow-up study *	23.60 ± 12.95 (6–71)
Age *	61.70 ± 9.51 (37–82)
Sex Female Male	19 (57.6%) 14 (42.4%)
Status Dead Alive	32 (96.7%) 1 (3.3%)
Location of primary tumor Pancreatic head Body Uncinated process (UP) Overlapping head/UP Overlapping head/body Overlapping body/tail Tail	15 (45.5%) 4 (12.1%) 4 (12.1%) 3 (9.1%) 3 (9.1%) 3 (9.1%) 1 (3.0%)
cT stage at diagnosis cT1 cT2 cT3 cT4	0 (0.0%) 6 (18.2%) 15 (45.5%) 12 (36.3%)
cN stage at diagnosis cN0 cN1 cN2	25 (75.8%) 6 (18.1%) 2 (6.1%)
cM stage at diagnosis cM0 cM1	33 (100%) 0 (0.0%)
Histology Adenocarcinoma	33 (100%)
Duodenal infiltration No Dubious Yes	31 (93.9%) 2 (6.1%) 0 (0.0%)

* Results expressed as mean ± SD (range).

**Table 2:** Resectability and pathological results.

Characteristic	Number of Patients (%)
**Radiological post-neoadjuvant resectability-Clinical borderline group (n = 13)**	
Underwent surgery and was completed	8 (61.5%)
Underwent surgery and was cancelled #	2 (15.4%)
Died before surgery	1 (7.7%)
No response or PD	2 (15.4%)
**Radiological post-neoadjuvant resectability-Clinical unresectable group (n = 20)**	
Underwent surgery and was completed	3 (15.0%)
Response but not underwent surgery	5 (25.0%)
No response	12 (60.0%)
**Post-neoadjuvant surgery**	
Yes	13 (39.4%)
No	20 (60.6%)
**Possibility of pathological analysis in operated patients (n = 13)**	
Borderline group	10 (76.9%)
Yes	8 (80.0%)
No #	2 (20.0%)
Unresectable group	3 (23.1%)
Yes	3 (100.0%)
No	0 (0.0%)
ypT stage *	
ypT1	2 (18.2%)
ypT2	6 (54.5%)
ypT3	2 (18.2%)
ypT4	0 (0.0%)
ypTx	1 (9.1%)
ypN stage *	
ypN0	5 (45.4%)
ypN1	3 (27.3%)
ypN2	3 (27.3%)
Pathological response (TRS system) *†	
Score 0	0 (0.0%)
Score 1	3 (27.3%)
Score 2	6 (54.5%)
Score 3	2 (18.2%)
Resection margins *	
R0 surgery (tumour-free)	8 (72.7%)
R1 surgery (microscopic disease)	3 (27.3%)
R2 surgery (macroscopic disease)	0 (0.0%)

* Percentage of the total number of operated patients. # Surgery not completed due to intraoperative findings of hepatic metastases (n = 1) and gastric infiltration (n = 1). † Tumor Response Scoring (TRS): 0, no viable tumour cells; 1, single cells or rare small groups of cancer cells; 2, residual cancer with evident tumour regression; 3, extensive residual cancer with no evident tumor regression.

**Table 3:** Resectability after Neoadjuvant treatment regarding pretreatment tumour extension.

		Pretreatment		Total	*p*-Value
		BRPC	LAPC		
RESECTABILITY	Non-resectable	2(13.6%)	16 (80%)	18 (55.5%)	
	Resectable	11(86.4%)	4 (20%)	15 (45.5%)	*p* < 0.0001
		13(39.4%)	20 (60.6%)	33 (100.0%)	

## Data Availability

The data presented in this study are available upon request from the corresponding author due to ethical reasons.

## Abbreviations

Overall survival	OS
Chemotherapy	ChT
Radiotherapy	RT
Chemoradiotherapy	CRT
Borderline resectable pancreatic cancer	BRPC
Locally advanced pancreatic cancer	LAPC
Local control	LC
Local progression	LP
Stereotactic body radiotherapy	SBRT
Conventional fractionated radiotherapy	CFRT
Biological effective dose	BED
Organs at risk	OAR
Planned treatment volume	PTV
Standard linear accelerator	LINAC
Deep inspiration breath holding	DIBH
Computer tomography	CT
Magnetic resonance	MR
4D computed tomography	4D/CT
Gross tumor volume	GTV
Simultaneous integrated boost	SIB
Magnetic resonance-guided radiation therapy	MRgRT
Gy	Gray
Tumor Response Scoring	TRS
Stereotactic ablative radiotherapy	SABR
The Society of Abdominal Radiology/American Pancreatic Association	SAR/APA
Multidisciplinary tumor board	MTB
Classification system of the College of American Pathologists	CAP
Freedom from local progression	FFLP
Cancer-specific survival	CSS
Disease-free survival	DFS
Freedom from distant failure	FFDF
Median follow-up	mfup
Follow-up	FUP
